# Atypical Presentations of Acyclovir-Resistant Herpes Simplex Virus in Immunocompromised Patients: A Case Series

**DOI:** 10.7759/cureus.93215

**Published:** 2025-09-25

**Authors:** Keri Allen, Alan Wang, Matthew A Hibdon, Robert Castro, John Greene

**Affiliations:** 1 Medicine, Morsani College of Medicine, University of South Florida, Tampa, USA; 2 Osteopathic Medicine, Nova Southeastern University Dr. Kiran C. Patel College of Osteopathic Medicine, Clearwater, USA; 3 Infectious Diseases, Moffitt Cancer Center, Tampa, USA

**Keywords:** acute myeloid leukemia, antiviral resistance, hemophagocytic lymphohistiocytosis, herpes simplex virus, immunocompromised, necrotizing cutaneous lesions

## Abstract

Herpes simplex virus (HSV) infections in immunocompromised patients can have atypical presentations - anywhere from granulomatous to linear erosive lesions - and become refractory to standard therapy. We report three cases of necrotizing cutaneous HSV infection in immunocompromised patients with underlying hematologic malignancies or prior chemoradiation, all presenting with atypical and refractory manifestations of the disease. One case involved a 53-year-old female patient with relapsed/refractory acute myeloid leukemia (AML) who developed a necrotizing cutaneous HSV infection refractory to acyclovir and subsequently foscarnet. Two additional cases highlight similar cutaneous presentations of HSV, a patient with anal squamous cell carcinoma and a patient with multiple myeloma, underscoring the variability and severity of the disease in the setting of profound immunosuppression. These cases underscore the possible need for prolonged antiviral therapy, early resistance testing, and adjunctive treatment strategies in managing atypical HSV infections in severely immunocompromised patients. Further studies are needed to establish evidence-based protocols for such challenging cases.

## Introduction

Herpes simplex viruses (HSV-1 and HSV-2) are enveloped, double-stranded DNA viruses responsible for a wide range of disease manifestations worldwide. The characteristic skin lesions of HSV appear as grouped, fluid-filled vesicles with underlying erythema. These lesions are often described as "dew drops on a rose petal," and in immunocompetent hosts, the infection is often self-limited or even asymptomatic [1]. In fact, many infections occur without recognized symptoms of herpes disease, highlighting the challenge of controlling HSV transmission, as many individuals are unaware of their status.

Clinical diagnosis can be difficult as well, as lesions are self-limited and may be absent at the time of clinical evaluation [1]. However, if lesions are present, diagnosis should be confirmed with type-specific virologic testing (nucleic acid amplification test (NAAT) or culture) [1]. Polymerase chain reaction (PCR) testing is also crucial for identifying HSV DNA in cerebrospinal fluid, enabling accurate and timely diagnosis of herpetic meningitis, commonly caused by HSV-2. However, current diagnostic tools have limitations: PCR sensitivity can vary depending on specimen type and timing, and resistance testing, whether genotypic or phenotypic, is not widely available in real time, often delaying therapeutic adjustments.

In contrast, immunocompromised individuals may develop prolonged, atypical, and sometimes necrotizing manifestations of HSV [1]. These unusual forms are often driven by antiviral resistance mechanisms, particularly thymidine kinase (TK) mutations and alterations in viral DNA polymerase, which reduce the efficacy of standard therapies like acyclovir. Consequently, cutaneous manifestations can include chronic mucocutaneous HSV infections with wart-like or ulcerative lesions that are resistant to standard antiviral therapies [2]. Other cutaneous patterns that can be observed include granulomatous vasculitis and anogenital lesions [3]. Additionally, visceral involvement can include dissemination to the liver, adrenals, lungs, and brain, particularly in those with advanced immunosuppression [4].

There have been reports of HSV resistance to standard antiviral treatments in immunocompromised individuals, potentially linked to long-term antiviral therapy and sustained high levels of viral replication that arise from ineffective immune responses [5]. Therefore, clinicians need to adopt a tailored approach to manage HSV infections in this vulnerable patient population. In such patients, the standard short-course antiviral therapy (acyclovir, valacyclovir, or famciclovir for typically one-two weeks) may be insufficient [2]. Instead, a more aggressive and prolonged treatment strategy, guided by early resistance testing and, if necessary, the use of alternative agents such as foscarnet, could be required [2,6]. This report presents three cases of immunocompromised patients who presented with atypical cutaneous HSV infections. These patients highlight the challenges of managing such infections in the setting of profound immunosuppression and an aberrant inflammatory response.

## Case presentation

Case 1

**Figure 1:**
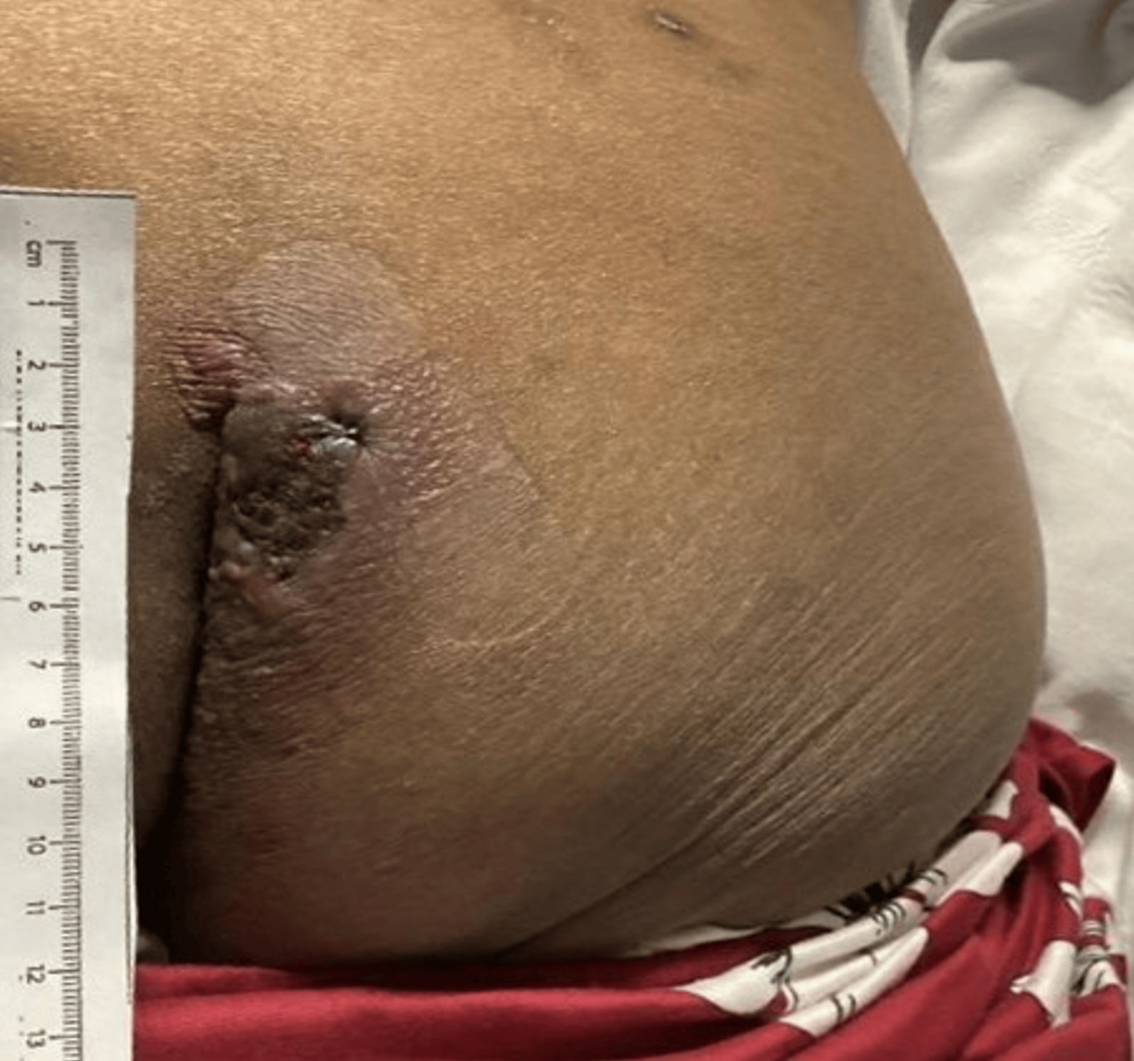
The indurated, tender plaque on the right buttock was present on admission. Punch biopsy revealed leukocytoclastic vasculitis with fibrin thrombi, early vascular necrosis, and viral cytopathic changes; immunostaining was positive for HSV-1/2. The vasculitic pattern underscores HSV’s capacity to trigger immune-mediated vascular injury, a complication reported in immunocompromised hosts. Importantly, such vascular involvement may serve as an early histopathological marker of antiviral resistance in atypical presentations, highlighting the need for heightened clinical suspicion and timely resistance testing. HSV: Herpes simplex virus

A 53-year-old female patient with relapsed/refractory acute myeloid leukemia (AML) and a history of multiple chemotherapy regimens and an allogeneic hematopoietic cell transplant, was admitted with neutropenic fever. Her recent treatment included azacitidine, venetoclax, and gilteritinib, with a bone marrow biopsy revealing 40-50% blasts. Shortly after admission, she received repeat induction chemotherapy with CLAG (cladribine, cytarabine, and Granulocyte Colony-Stimulating Factor (G-CSF)) combined with venetoclax. Notably, on admission she also presented with an indurated tender plaque on her right buttock that had persisted for three weeks (Figure 1). Further evaluation revealed the lesion to be associated with cellulitis and an underlying presacral/perianal abscess, raising concerns for an atypical infection manifestation in this profoundly immunocompromised host.

**Figure 2:**
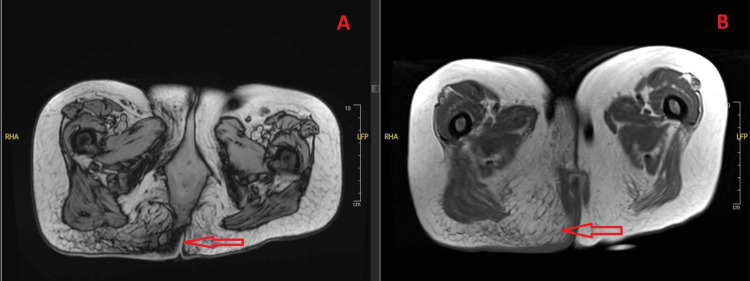
Axial pelvic MRI (A) Axial T2-weighted MRI demonstrates a presacral/perianal fluid collection with peripheral rim hyperintensity (arrow), consistent with an abscess, extending into the right gluteal region. Associated surrounding fat stranding and soft tissue edema are also evident; (B) A more caudal axial slice shows further extension of the fluid collection into the perianal soft tissues (arrow), with asymmetric involvement of the right buttock, correlating with the site of the patient’s ulcerated plaque.

A detailed diagnostic workup was initiated early in her hospital course. On the day of admission (Day 0), CT imaging of the thorax, abdomen, and pelvis showed mesenteric fat stranding (suggestive of ileitis), gluteal cleft thickening, inguinal lymph node enlargement, ground-glass opacities in the lung, and abdominal wall nodules. MRI of the pelvis performed the same day further delineated the presacral abscess and its extension into adjacent soft tissues (Figure 2).

**Figure 3:**
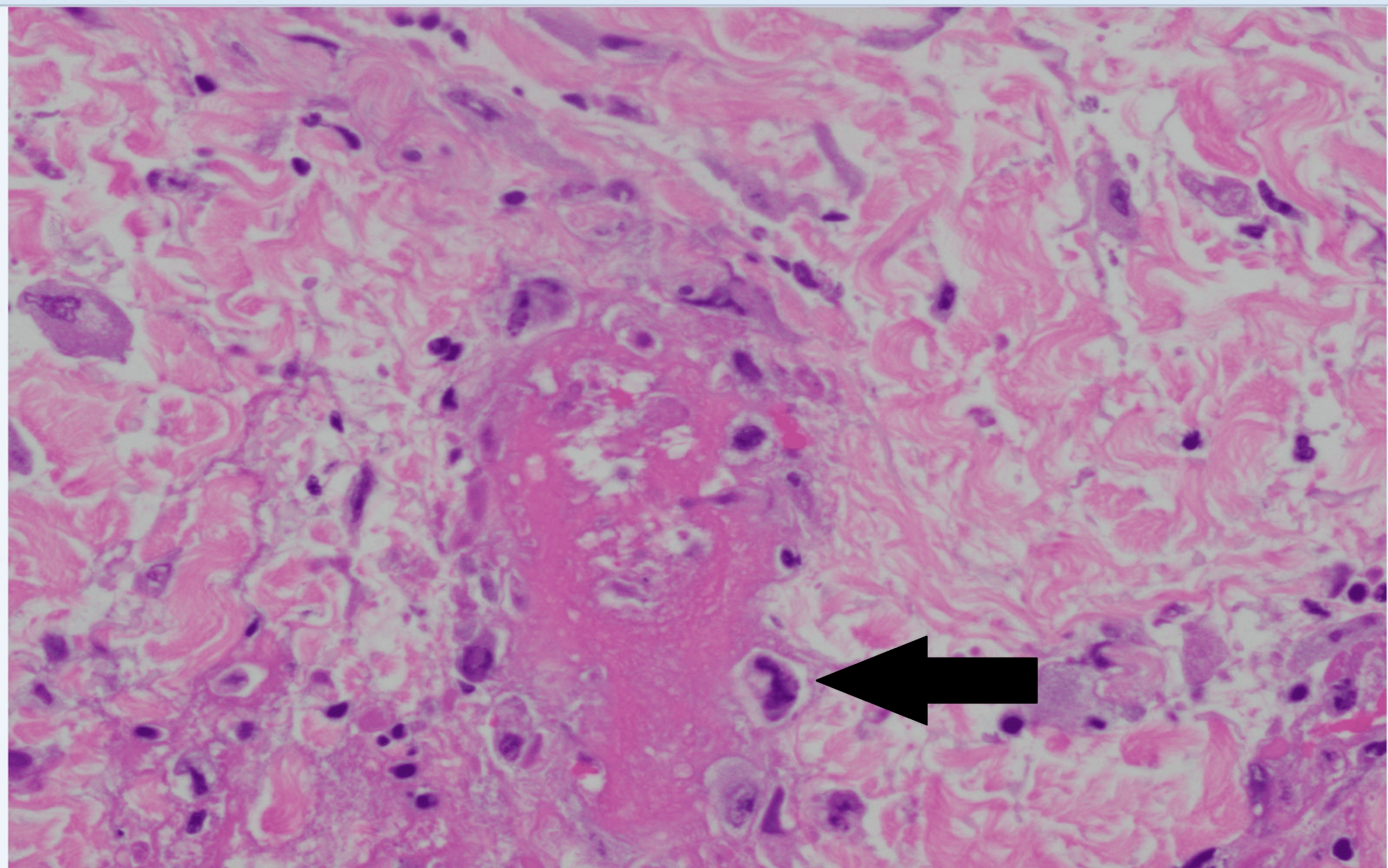
Histopathology of right buttock biopsy H&E staining shows a thrombosed vessel (arrow) with surrounding necrosis and viral cytopathic changes in endothelial cells, characterized by nuclear enlargement, chromatin margination, and multinucleation, consistent with HSV infection. H&E: Hematoxylin and eosin; HSV: Herpes simplex virus

**Figure 4:**
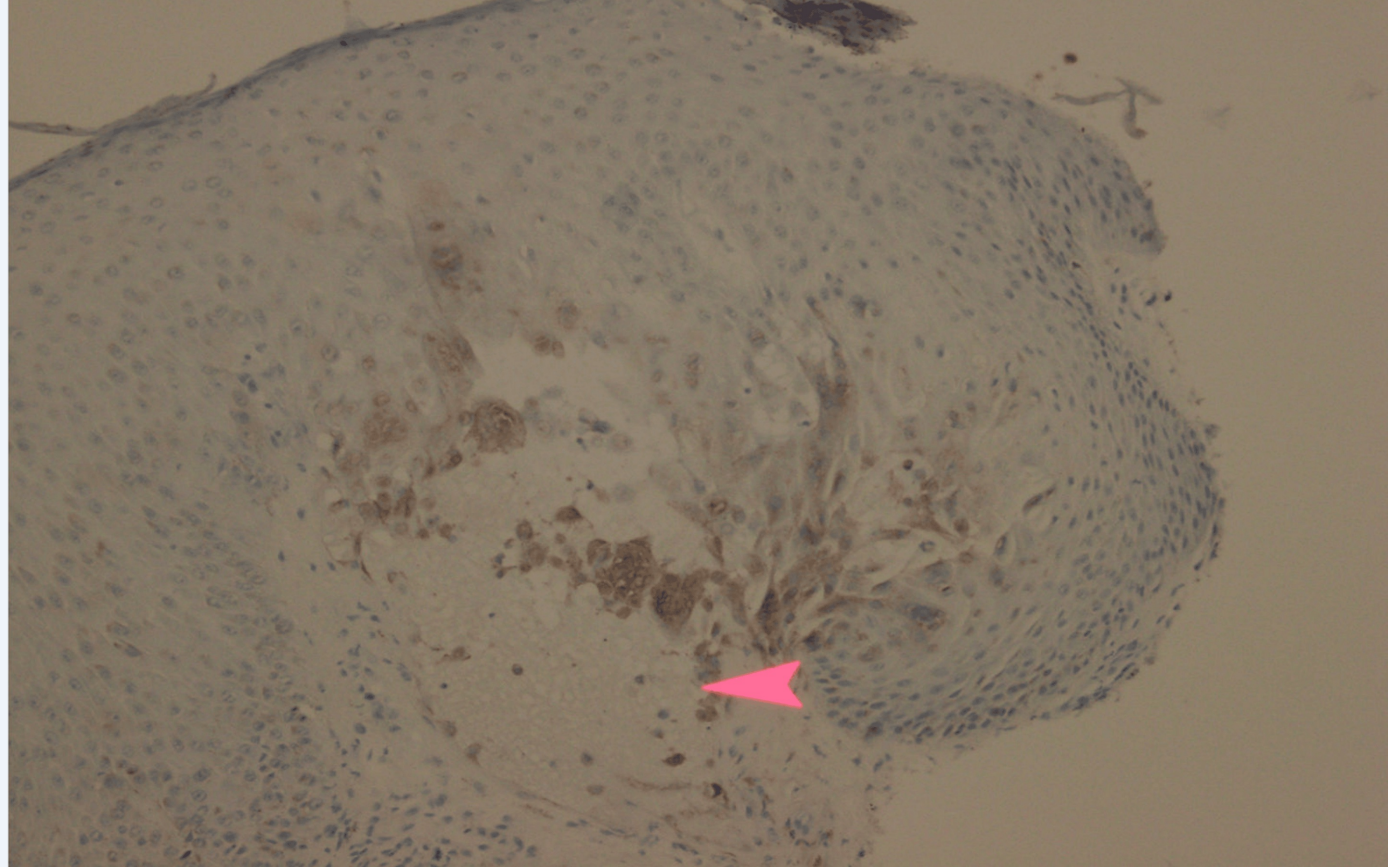
Immunohistochemical staining for HSV Biopsy of the right buttock lesion demonstrates HSV antigen positivity in epidermal cells, highlighted by brown nuclear and cytoplasmic staining. The arrow indicates HSV-positive cells, confirming HSV infection. HSV: Herpes simplex virus

**Figure 5:**
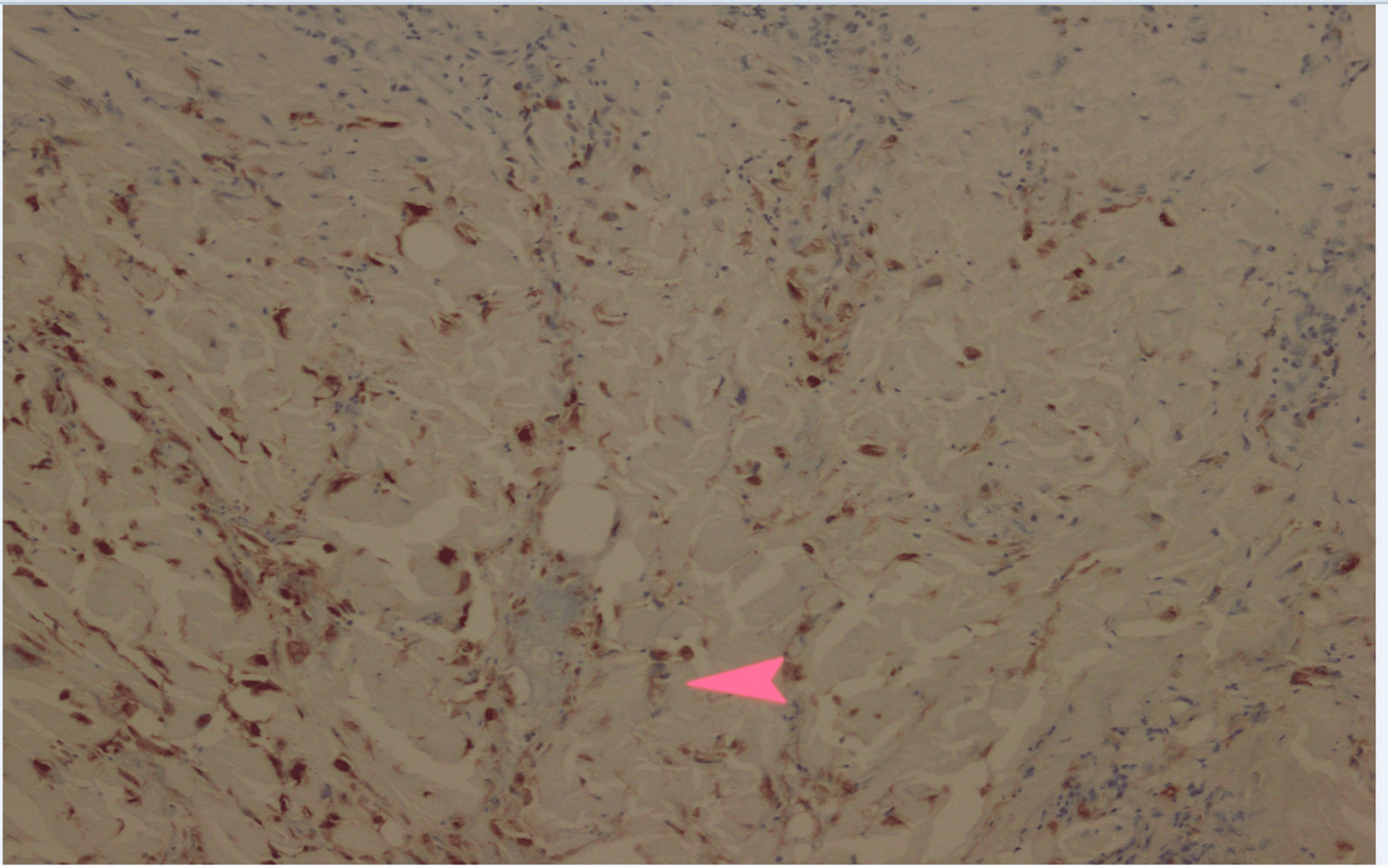
Immunohistochemical staining for HSV in dermis Immunohistochemistry demonstrates HSV antigen positivity in dermal cells, highlighted by brown nuclear and cytoplasmic staining. The arrow indicates HSV-positive cells, confirming viral involvement beyond the epidermis. This finding supports widespread cutaneous HSV infection with extension into deeper tissue layers. HSV: Herpes simplex virus

The following day (Day 1), wound cultures from the gluteal cleft grew *Staphylococcus epidermidis* and *Enterococcus faecalis*. On Day 2, a dermatologic punch biopsy of the right buttock lesion was performed and demonstrated leukocytoclastic vasculitis with fibrin thrombi, early vascular necrosis, and viral cytopathic changes. Immunostaining confirmed HSV-1/2 infection, while stains for cytomegalovirus (CMV) and fungi (Periodic Acid-Schiff (PAS)/Grocott's Methenamine Silver (GMS)) were negative (Figures 3, 4, 5). Two days later, to manage the cutaneous HSV infection, valacyclovir was initiated and planned for a one-week course.

**Figure 6:**
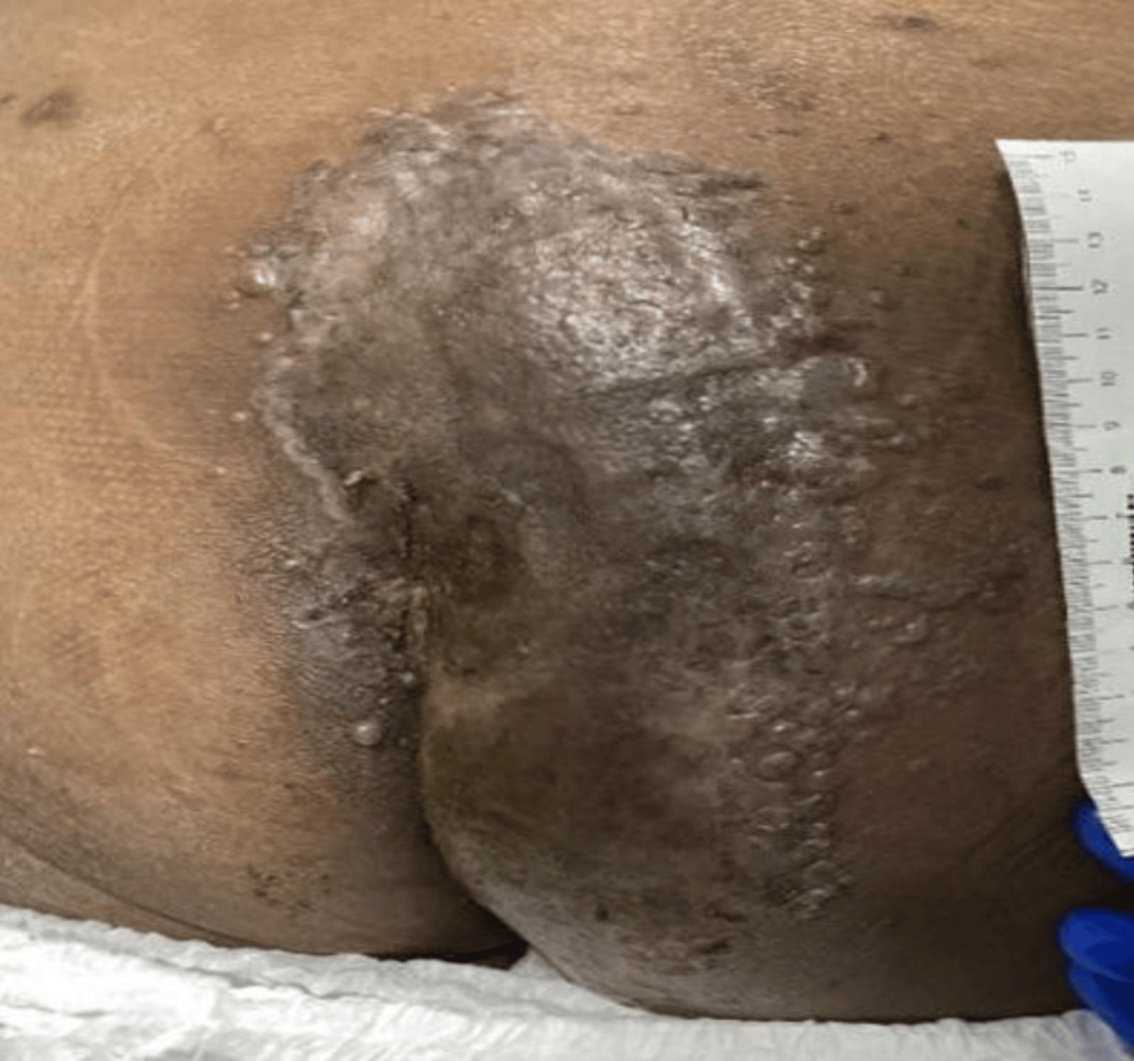
As the patient’s infection persisted with worsening gluteal myositis and concerns for necrotizing fasciitis, the antimicrobial regimen was escalated on Day 9 to meropenem and linezolid.

As her infection persisted with worsening gluteal myositis and concerns for necrotizing fasciitis (Figure 6), the antimicrobial regimen was escalated to meropenem and linezolid. Additionally, 26 days into her hospital stay, vesicle HSV PCR returned positive, confirming active HSV involvement. Thus, valacyclovir was resumed for continued HSV management.

Over the next week, the patient displayed persistent HSV activity and concerns for antiviral resistance, prompting the team to escalate therapy to IV foscarnet. The patient’s recurrent fevers, worsening cytopenias, and markedly elevated inflammatory markers - most notably a ferritin of 595,563 ng/mL, fibrinogen of 290 mg/dL (within normal limits), CD25 of ~3,000 U/mL, and fasting triglycerides of 206 mg/dL - raised concerns for a hemophagocytic lymphohistiocytosis (HLH)-like process that may have further driven tissue necrosis.

Despite extensive antimicrobial and antiviral therapy and robust supportive care (including growth factors, transfusions, and tumor lysis prophylaxis), the patient’s condition continued to deteriorate. Ultimately, a decision was made to transition to comfort measures only, and she was ultimately discharged to hospice care.

The entire clinical course, diagnostic findings, and therapeutic escalations are displayed in Table 1.

**Table 1:** Clinical course, diagnostic findings, and therapeutic escalations HSV: Herpes simplex virus; EEG: Electroencephalogram; PCR: Polymerase chain reaction; EBV: Epstein–Barr virus

Hospital Day	Event	Therapy
0	Admission: fever + buttock plaque; CT/MRI shows abscess/myositis	Clindamycin → cefepime + metronidazole + vancomycin
1	Wound cultures: *S. epidermidis, Enterococcus*	Broad-spectrum antibiotics continued
2	Biopsy: leukocytoclastic vasculitis, HSV-1/2 confirmed	Valacyclovir started
4	Switched to Zosyn	Zosyn (Days 4-9)
9	Worsening lesion; escalation to meropenem + linezolid	Meropenem + linezolid
14	EEG diffuse slowing; lumbar puncture normal (no CNS infection)	Continued antimicrobials
20	Blood cultures: *S. epidermidis*	Vancomycin; restarted meropenem
23	MRI: worsening myositis/abscess; surgery consulted; central line removed	Zosyn maintained
26	HSV PCR positive; valacyclovir resumed	Valacyclovir resumed
29	EBV PCR 14,500 copies/mL	Supportive care, monitoring
33	Blood cultures: *Candida glabrata*	Meropenem + antifungal coverage
34	Escalation to IV acyclovir, then foscarnet; micafungin started	Acyclovir → foscarnet; micafungin
35	Goals-of-care discussion; transition to comfort care	Comfort care measures
37	Discharged to hospice	Hospice

Case 2

A 72-year-old woman with a history of chronic diarrhea and anal squamous cell carcinoma, previously treated with 5-fluorouracil, mitomycin, and radiation therapy, presented with anal pain and a newly developed perianal ulcer. She was febrile to 100.7°F and profoundly neutropenic, anemic, and thrombocytopenic (see Table 2 for admission laboratory results). Due to her immunocompromised state, broad microbiologic testing was initiated. Stool cultures grew *Pseudomonas aeruginosa*, blood cultures were negative, and urine cultures showed mixed bacterial flora. In response, cefepime and voriconazole were started to cover gram-negative and fungal pathogens. Contrast-enhanced CT imaging of the abdomen and pelvis revealed anorectal wall thickening, likely related to prior radiation therapy, and soft tissue thickening in the perianal region, suggestive of an infectious or inflammatory process.

**Table 2:** Admission laboratory results WBC: White blood cells

Test	Result	Reference Range
WBC	0.48 × 10³/µL	4.0–11.0 × 10³/µL
Bands	21%	<10%
Hemoglobin	7.3 g/dL	12.0–16.0 g/dL
Platelets	26 × 10³/µL	150–450 × 10³/µL

**Figure 7:**
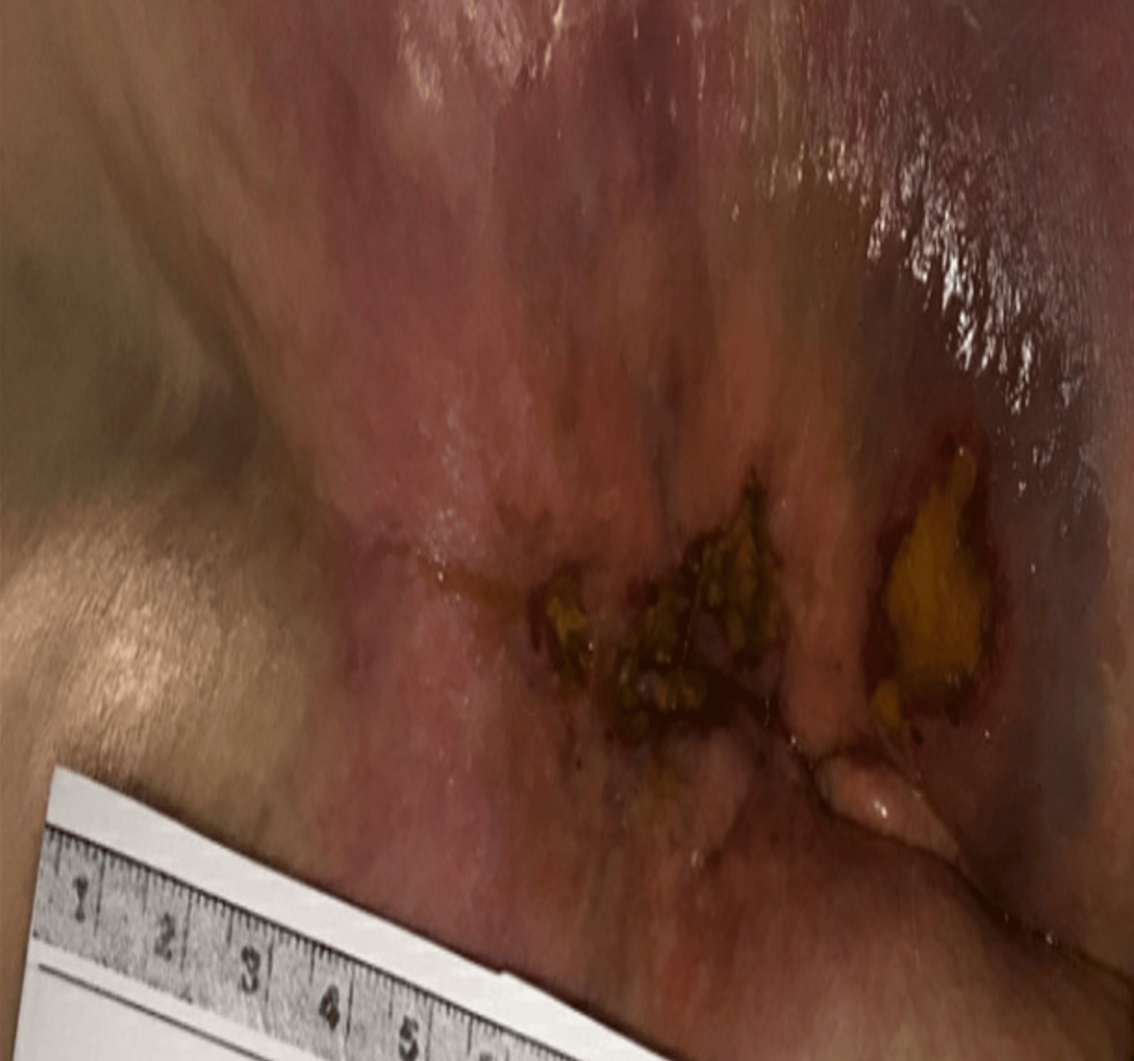
Two irregular perianal ulcers were measured at approximately 3.5 cm and 2.0 cm in length. Both lesions have shallow, erythematous borders with yellowish slough and fibrinous exudate at the base. The surrounding skin appears inflamed with areas of violaceous discoloration. HSV PCR testing from the ulcer biopsy returned positive, confirming HSV infection. HSV: Herpes simplex virus; PCR: Polymerase chain reaction

On physical exam, she had a painful, ulcerative lesion in the perianal area with irregular borders and erythematous surrounding skin (Figure 7). Given her prior chemoradiation, it is plausible that radiation-induced mucosal changes compromised local tissue integrity and altered immune surveillance, thereby predisposing her both to HSV reactivation and to atypical lesion morphology in the setting of profound cytopenias.

Given the clinical suspicion for viral reactivation, particularly HSV, a biopsy of the lesion was obtained for PCR testing and returned positive, confirming HSV as the underlying etiology. She was started on oral valacyclovir 1 gram twice daily, with close monitoring of her immunosuppressive status and wound progression.

Case 3

**Figure 8:**
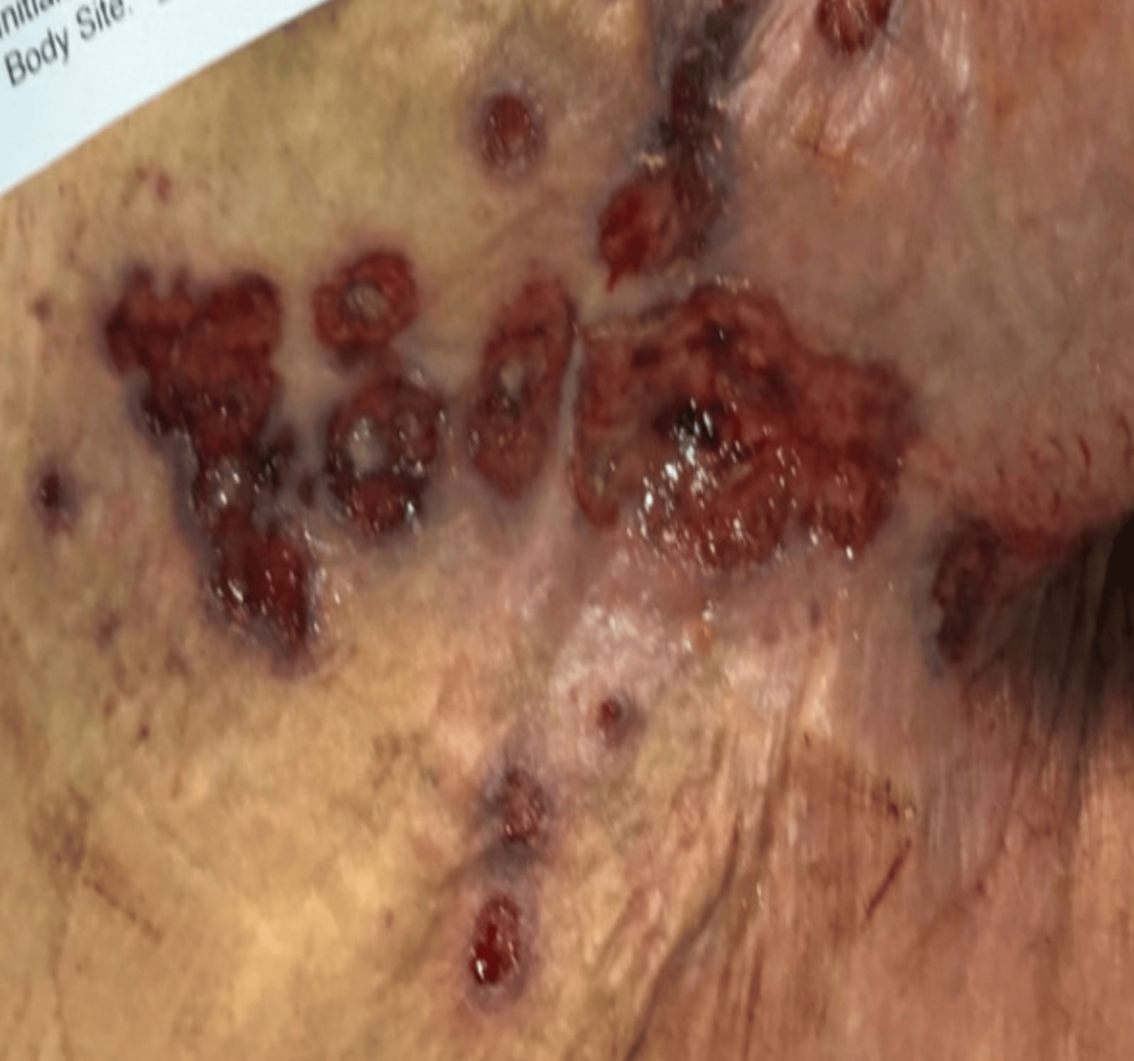
The severe ulcerations exhibited multiple irregular, coalescing ulcers over the coccyx and upper buttock, characterized by red, moist bases and yellowish slough. Surrounding skin appears erythematous and inflamed, with signs of maceration.

Similarly, a 63-year-old woman with a history of kappa light chain multiple myeloma, previously treated with multiple lines of therapy, presented with progressive fatigue and increased pain at the site of a sacral ulcer that had been developing over the prior two-three weeks. Her medical history was also notable for pancytopenia, lymphopenia, and extensive lytic bone lesions with lumbar compression fractures. She endorsed poor appetite, but denied experiencing fever, chills, or night sweats. On examination, there was a cluster of locally scattered, ulcerative, bleeding lesions over the coccyx and upper buttock region (Figure 8).

The ulcers were irregular in shape with moist, erythematous bases, yellowish slough, and focal areas of necrosis. The surrounding skin was inflamed, with evidence of maceration and superficial tissue breakdown. In an immunocompromised host, this morphology raised a broad differential that initially included pyoderma gangrenosum, pressure-related injury, and possible fungal superinfection, in addition to viral etiologies. Given the chronicity, her underlying immune suppression, and clinical experience with similar cases, a biopsy of the lesion was sent for HSV PCR testing. Empiric intravenous acyclovir was initiated while awaiting results.

## Discussion

Case 1 highlights an atypical necrotizing cutaneous HSV infection in a profoundly immunocompromised patient with relapsed AML. The patient’s extensive necrotic perianal and cutaneous lesion, refractory to multiple courses of acyclovir-based regimens, underscores the challenges of managing HSV in the setting of prolonged neutropenia and immunosuppression and, most importantly, the need for early and accurate diagnosis. Cases 2 and 3 further illustrate how HSV can cause similar necrotizing lesions in other immunocompromised settings. In Case 2, the patient had prior chemoradiation for anal squamous cell carcinoma, and in Case 3, an elderly patient with multiple myeloma presented with chronic sacral ulcers and HSV-positive lesions. These cases demonstrate that atypical HSV can occur across a broad spectrum of immunosuppressive backgrounds.

The typical course of HSV infection in immunocompetent hosts involves a 1-2-week course of acyclovir or valacyclovir for treatment. However, in those who are immunocompromised, HSV may not only become resistant to standard therapies but also manifest with atypical clinical features. A summary table (Table 3) is provided to outline the spectrum of atypical HSV presentations, which further emphasizes the varied and challenging manifestations of HSV in immunocompromised hosts.

**Table 3:** Summary of atypical presentations of HSV HIV: Human immunodeficiency virus; HPV: Human papillomavirus; HSV: Herpes simplex virus; PCR: polymerase chain reaction; IBD: Inflammatory bowel disease

Presentation	Clinical Description	Associations	Comments	Recommended Initial Diagnostic Test	Typical Antiviral Response	Reference
Granulomas	Granulomatous inflammation, mimicking other chronic granulomatous diseases.	Rare; typically seen in immunocompromised patients.	Diagnosis may be delayed due to overlapping features with other granulomatous dermatoses.	Biopsy with histopathology ± PCR	Often resistant; may require escalation to foscarnet	[3]
Vasculitis	Leukocytoclastic vasculitis with fibrin deposition and necrosis.	Reported in immunocompromised individuals.	May resemble autoimmune vasculitis; biopsy is key for diagnosis.	Punch biopsy with HSV PCR and immunostaining	Variable; resistance common, foscarnet often needed	[7]
Necrotizing Lesions	Cutaneous ulcerations with necrosis, mimicking pyoderma gangrenosum or ecthyma gangrenosum.	Can occur especially in immunocompromised patients.	Misdiagnosis can lead to inappropriate treatment and severe consequences.	Tissue biopsy + culture ± PCR	Frequently acyclovir-refractory; foscarnet typically required	[8]
Linear Erosive Lesions	Known as the "knife-cut" sign; deep, linear fissures in skin folds in immunocompromised patients, mimicking the ulcers associated with metastatic Crohn’s disease.	Classically seen in severely immunocompromised patients, can also involve patients with chronic illnesses (chronic kidney disease, type 2 diabetes, morbid obesity) and acute systemic illnesses.	Indicative of severe tissue compromise. Delayed diagnosis can lead to delayed treatment and potentially adverse outcomes.	Biopsy with HSV PCR; rule out Crohn’s/IBD	Mixed response; acyclovir may work, but resistance possible	[5]
Herpes Vegetans	Exophytic, verrucous, wart-like, proliferative lesions.	Common in HIV or severely immunocompromised patients.	It is crucial to differentiate herpes vegetans from other verrucous lesions (i.e., squamous cell carcinoma or HPV-related lesions) in the anogenital area.	Biopsy with immunostaining ± HPV testing	Frequently acyclovir-resistant; foscarnet often required	[9]
Eczema Herpeticum	Sudden, painful, and widespread rash with vesicles, pustules, and erosions accompanied by fever.	Atopic dermatitis is a major predisposing factor.	Dermatologic emergency; requires immediate systemic antiviral therapy.	Lesion swab for HSV PCR ± Tzanck smear	Usually responsive to acyclovir if treated promptly	[10]

These atypical presentations require a high index of suspicion and appropriate diagnostic testing to ensure accurate diagnosis and management. Notably, the Karius test played a crucial role in our diagnostic process, confirming HSV with a reported level of 271,355 copies/mL. This emerging technology is proving invaluable for diagnosing obscure and atypical infections within the herpes virus family, particularly in complex cases such as this [11]. From a management standpoint, whether to pursue an early empirical switch to foscarnet in select high-risk patients or to await confirmatory genotyping remains a point of clinical debate, underscoring the need for individualized decision-making based on patient risk factors and institutional resources.

In July 2024, Shafat et al. underscored the urgent need for a standardized definition of refractory HSV infection, noting that the lack of consensus complicates clinical management and delays appropriate escalation of therapy [6]. This ambiguity also poses a major barrier to consistent patient enrollment in clinical trials. To address this gap, Chemaly et al. published a literature review in January 2025, proposing definitions for key clinical terms, including refractory HSV infection, recurrent HSV infection, breakthrough recurrent HSV infection, and antiviral-resistant HSV infection, for use in clinical practice and research settings [12]. For example, at least one of the following two criteria must be met to be considered a refractory HSV infection: 1) the lack of clinical improvement in HSV-positive mucocutaneous lesion(s) after at least seven days of appropriately dosed, directed anti-HSV therapy, in the absence of other plausible causes of mucositis, such as recent high-dose chemotherapy, irradiation, oral GVHD, fungal or other viral infections; or 2) the occurrence of a new HSV-positive mucocutaneous lesion(s) after receiving appropriately dosed, directed anti-HSV therapy for at least seven days (excluding prophylaxis and suppressive antiviral therapy) [12].

These definitions aim to guide diagnostic and therapeutic decision-making while supporting better trial design and compatibility of outcomes.

Another important consideration is resistance to acyclovir and other nucleoside analogs, which are most often associated with mutations in viral TK (UL23) or DNA polymerase (UL30), impairing the drug’s activation or incorporation into viral DNA [13]. The proposed definition for antiviral-resistant HSV infection is “a refractory HSV infection… in addition to a viral genetic alteration(s) that decrease(s) susceptibility and/or phenotypic assay demonstrating increased EC50 above the assay cutoffs to 1 or more antiviral drugs” [12]. In immunocompetent individuals, the prevalence of acyclovir resistance is low, ranging from 0.1% to 0.7% [14]. However, in immunocompromised patients, particularly those with impaired T-cell-mediated immunity, prolonged antiviral exposure, suboptimal dosing of acyclovir, or ongoing viral replication, the risk is significantly higher [13,14]. Among hematopoietic stem cell transplant (HSCT) recipients, HSV infections remain a clinically significant complication, with reported prevalence rates of refractory and resistant (R/R) HSV infection ranging from 0% to 14%, and some studies reporting rates as high as 46% [6,13]. R/R HSV infections in HSCT recipients are associated with prolonged antiviral therapy, recurrent infections, renal failure, and increased mortality [6]. Given these risks, it is crucial to perform early HSV resistance testing in patients who fail to respond to first-line antiviral therapy. In the cases presented, the lack of resistance testing may have delayed escalation to second-line agents such as foscarnet or cidofovir, potentially impacting the patient’s outcome.

When treating such infections, in accordance with the United States Centers for Disease Control and Prevention (CDC) guidelines, we emphasize that treatment courses for genital herpes in immunocompromised patients, such as those with HIV, may need to be extended, potentially weeks or months, until lesion resolution is achieved [1]. This is especially important because immunocompromised patients can experience prolonged or severe episodes of HSV, and lesions might recur or persist despite standard antiviral therapy. In the case presented, insufficient duration and aggressiveness of antiviral therapy likely contributed to the progression of the large necrotizing area. The National Comprehensive Cancer Network (NCCN) guidelines recommend foscarnet as the treatment of choice for acyclovir-resistant HSV infections, although it is associated with significant nephrotoxicity and requires intensive monitoring [6]. Other current treatment options for R/R HSV infections include IV cidofovir and high-dose IV acyclovir [6].

Given the growing prevalence of antiviral resistance and the limitations of currently approved therapies, adjunctive and emerging treatment options warrant careful consideration, particularly in immunocompromised populations. In all three cases, prolonged HSV lesions in the setting of immunosuppression necessitated careful virologic evaluation, a high index of suspicion for resistance, and consideration of extended or adjunct antiviral therapy. Several promising strategies-including topical antivirals, immunomodulatory agents, and novel antiviral compounds-have been explored to enhance therapeutic efficacy in R/R HSV infections. We encourage the use of adjunctive measures, such as compounding a topical antiviral paste (using trifluridine with Aquaphor) alongside IV therapy. Trifluridine, an antiviral agent effective against HSV, acts independently of viral TK, making it a potential alternative for acyclovir-resistant strains. Yacoub et al. suggest a combined regimen of topical trifluridine and oral famciclovir for treating cutaneous herpes in immunocompromised patients, particularly those with potential acyclovir resistance [5]. Another approach, such as the adjunctive use of interferon-α (IFN-α), has shown promise. IFN-α's synergistic antiviral effect with acyclovir stems from alterations in nucleoside metabolism, creating a more favorable environment for acyclovir to compete with natural substrates for viral enzymes, enhancing its antiviral activity [15]. However, the use of IFN-α is limited by its high cost and limited availability in the United States, despite its availability in Europe. Pritelivir, a helicase-primase inhibitor, is currently under review and has shown promise in clinical trials for the treatment of R/R HSV infections [6]. In immunocompromised patients, pritelivir has demonstrated efficacy in the treatment of acyclovir-resistant HSV infections, with partial responses observed by Week 1 and complete responses by Week 4 of treatment [6]. In contrast to current treatments, such as foscarnet or cidofovir - both significantly associated with nephrotoxicity - this emerging therapy not only addresses the limitations of current treatments but offers hope for managing R/R HSV infections in severely immunocompromised patients. In the case presented here, such adjunctive measures may have benefited the patient.

Limitations of this report include its three-case design, the absence of comprehensive HSV resistance testing, and the presence of multiple confounding factors (e.g., concurrent bacteremia and candidemia) that may have influenced the overall outcome. While commercial and some academic reference laboratories offer genotypic and phenotypic resistance testing, these assays are not universally available in real time, which limited our ability to confirm resistance during the patients’ clinical course. Future studies should investigate through randomized controlled trials refractory/resistant HSV infection treatment outcomes in severely immunocompromised cancer patients.

## Conclusions

These three cases underscore the unique challenges in managing atypical, necrotizing HSV infections in immunocompromised patients. Given that necrotic lesions in this population may result from multiple etiologies, clinicians should maintain a broad differential that includes entities such as ecthyma gangrenosum, particularly in neutropenic individuals, to ensure timely and appropriate therapy. Early resistance testing coupled with extended antiviral regimens and escalation to agents such as foscarnet or even IFN-α when indicated may be warranted to achieve resolution. The complex interplay between aberrant inflammatory responses, persistent viral activity, and prolonged neutropenia highlights the need for tailored management strategies. To improve outcomes, standardized care pathways for atypical HSV in cancer and transplant patients, modeled after existing protocols for invasive fungal infections or CMV, should be developed to guide clinicians in early recognition, resistance testing, and therapeutic escalation. Future guidelines should also consider incorporating earlier genotypic resistance testing in high-risk patients, which could enable more timely transitions to second-line antivirals and potentially improve survival.
